# Editorial: Insights in veterinary experimental and diagnostic pathology: 2021

**DOI:** 10.3389/fvets.2022.1075611

**Published:** 2022-11-29

**Authors:** Francisco Javier Salguero

**Affiliations:** United Kingdom Health Security Agency Porton Down, Salisbury, United Kingdom

**Keywords:** pathology, molecular diagnosis, diagnostics, experimental, editorial

Veterinary pathology is a rapid changing discipline within Veterinary Sciences. The role of the pathologist within diagnostic and research studies has moved from a very subjective input to a more objective approach using new tools at hand, such as molecular techniques, digital image analysis and quantitative pathology (1, 2). The present Research Topic is focused on insights in Veterinary Experimental and Diagnostic Pathology, with four original research articles and a case report on a novel pathogen.

Larenas-Muñoz et al. reviewed the role of classic histopathology in the diagnostic of bovine tuberculosis, in combination with other tools as bacterial culture, PCR and serology. Histopathology continues to be a very valuable tool to monitor this disease, in cattle but also in other livestock and wildlife species. They discussed some of the advantages of pathology with a focus on the ability to characterize and categorize the lesions observed in infected animals.

Thaiwong et al. described the expression of two markers (carboxypeptidase A3 and tryptase) for lymph node metastasis in the very common canine mast cell tumors. The deception of metastasis in these tumors is crucial for the clinical management of the affected dogs. They established a reliable and highly sensitive molecular technique to detect the mRNA expression of mast cell-specific genes within lymph node tissue, a very valuable tool to detect and classify metastasis.

Following on the application of molecular techniques to study neoplasia in animals, Tekavec et al. described interesting aspects of canine nerve sheath tumors. This neoplasia is not studied in depth and the classification is not yet fully established as it happens with other tumors of the nervous tissue. They described a loss of expression of H3K27me3 in a subset of nerve sheath tumors but with no significant association with other histopathological features.

Luttman et al. developed a new technique to be used for DNA profiling and paternity testing in horses suing tetranucleotide and pentanucleotide short tandem repeat polymorphisms. Using this technique, they described a 17-plex panel of markers for the horse with very promising application in 16 different breeds and crossbred horses.

Finally, Anderson et al. described a case of *Paecilomyces formosus* infection in a dog. This anamorphic fungal agent rarely produces clinical disease in immunocompromised and immunocompetent animals. The case describes the clinical disease with a fatal outcome and the pathological analysis showing the dissemination of the fungus and the produced necrotic foci in different organs. They reported for the first time the disseminated peacilomycosis in a dog with the identification of the pathogen.

## Author contributions

The author confirms being the sole contributor of this work and has approved it for publication.

## Conflict of interest

The author declares that the research was conducted in the absence of any commercial or financial relationships that could be construed as a potential conflict of interest.

## Publisher's note

All claims expressed in this article are solely those of the authors and do not necessarily represent those of their affiliated organizations, or those of the publisher, the editors and the reviewers. Any product that may be evaluated in this article, or claim that may be made by its manufacturer, is not guaranteed or endorsed by the publisher.
